# Assessment of Seed Viability Before and After Storage in Forage Pea (*Pisum sativum* L. var. arvense) Using Field and Laboratory Methods

**DOI:** 10.3390/plants14182872

**Published:** 2025-09-15

**Authors:** Serhat Akyüz, Emine Serap Kizil Aydemir, Serkan Ateş

**Affiliations:** 1 Republic of Türkiye Ministry of Agriculture and Forestry Genotype Registration and Seed Certification Center, 06172 Ankara, Turkey; serhat.akyuz@tarimorman.gov.tr; 2 Department of Field Crops, Faculty of Agricultural and Natural Sciences, Seyh Edebali University, 11230 Bilecik, Turkey; 3 Department of Animal and Rangeland Sciences, Oregon State University, Corvallis, OR 97331, USA; serkan.ates@oregonstate.edu

**Keywords:** forage pea, seed storage, germination, electrical conductivity, field emergence, forage yield, seed vigor, *Pisum sativum* L. var. arvense

## Abstract

This study aimed to evaluate the effects of different storage conditions on seed viability, vigor, and agronomic performance in six forage pea (*Pisum sativum* L. var. arvense) cultivars: Uysal, Özkaynak, Kurtbey, Ürünlü, Taşkent, and Nany. The research was conducted under Manisa (Türkiye) field conditions during the 2021–2022 growing season, with supporting laboratory tests performed in Ankara. Seeds were evaluated before and after a three-month storage period under two conditions: room temperature and cold storage (5 °C, 60% RH). Laboratory analyses included germination percentage, germination speed, electrical conductivity (EC), and field emergence tests. Results revealed that cold storage significantly preserved seed vigor and viability, as indicated by lower EC values, higher germination and field emergence rates. Among cultivars, ‘Taşkent’ and ‘Nany’ demonstrated superior physiological seed quality, while ‘Uysal’ was more sensitive to adverse storage conditions. In field trials, cold-stored seeds produced taller plants, earlier flowering, and higher green and dry forage yields compared to room-stored seeds. The cultivar ‘Ürünlü’ stood out with the highest average forage yield. These findings underline the importance of genotype-specific responses and proper seed storage practices to maintain seed quality and optimize forage productivity in cool-season legumes.

## 1. Introduction

Recent climatic fluctuations, the reduction in arable land, escalating production input costs, socio-cultural shifts, and rural-to-urban migration threaten the resilience of agricultural systems globally and in Turkiye. These dynamics undermine the ability of farming systems to sustainably meet growing food demands and pose increasingly serious risks to global food security if not promptly addressed [1].

Animal-derived proteins are essential for balanced human nutrition. The recommended daily protein intake is approximately 70 g per person, ideally sourced equally from plant and animal origins. While plant-based protein intake in Turkiye is generally adequate, consumption of animal-based protein remains insufficient. Livestock-derived foods, especially from cattle and small ruminants, play a critical role in providing animal proteins. However, feed costs accounting for nearly 70% of total production expenses severely affect the profitability of livestock operations, leading to reduced production, higher consumer prices, and diminished access to animal-sourced proteins.

A key barrier to sustaining livestock production is the insufficient supply of high-quality roughage. In Türkiye alone, 2022 data revealed 23.846 million hectares of arable land, of which only 2.752 million hectares are devoted to forage cultivation, despite a raw roughage demand of approximately 71.28 million tons annually [2,3]. Thus, a significant quality and quantity gap persists in forage resources.

Although cultivation of forage crops has increased in recent years, shortfalls in quality and quantity remain persistent. Economically feasible alternatives that support ruminant digestion are therefore vital. Enhanced forage cultivation can alleviate pressure on overgrazed pastures, reduce fallow land, improve soil fertility and water retention, and foster healthier crop rotations—offering multiple ecosystem and agronomic benefits.

Forage pea (*Pisum sativum* L. var. arvense) is an annual legume forage species highly valued for its palatability, balanced nutrition, and adaptability to cool climates. It requires minimal nitrogen fertilization, contributes 50–150 kgN/ha to the soil via biological fixation, and produces clean stubble conducive to subsequent crops. In Türkiye, it is cultivated across Southeastern Anatolia and coastal regions during the winter season [4,5]. At full flowering, its forage contains ~20% crude protein, while the seeds hold 20–30% crude protein and are rich in lysine qualities that make them suitable alternatives to soybean in European ruminant diets [6]. Its dual role as forage and green manure underscores its agroecological importance.

Given these attributes, evaluating the vigor and performance of forage pea seed lots prior to sowing is critical. Seed lots with high laboratory germination rates may nonetheless exhibit poor field emergence due to variations in seed vigor influenced by storage conditions. Seed vigor, a concept encompassing longevity, rapid germination, and robust seedling establishment under suboptimal conditions, is a stronger predictor of field performance than germination alone [7,8]. Techniques such as germination speed index (GSI), mean germination time (MGT), and radicle emergence tests at low temperatures have proven effective in vigor assessment and in forecasting field emergence in forage species [9,10,11]. Storage conditions play a pivotal role in determining seed longevity. Legume seeds, including peas, deteriorate rapidly under unfavorable moisture and temperature regimes [7,12]. Molecular studies show that oxidative damage, membrane degradation, and impaired DNA repair mechanisms underlie seed aging [7,13]. Modern research has also revealed that seed coat structure, antioxidant capacity, oligosaccharide composition, and genotype-specific traits modulate storability [7,14,15].

Despite extensive research on seed longevity across crops, relatively few studies focus on forage pea vigor and field emergence under real-world storage and environmental conditions. This study aims to address this gap by evaluating vigor, field emergence, and storage resilience in forage pea seed lots using standardized laboratory assays and field trials.

## 2. Materials and Methods

This study was conducted during the 2021–2022 growing season under the ecological conditions of Manisa, Türkiye, specifically on the experimental fields of the Beydere Seed Certification and Testing Directorate. Complementary laboratory analyses were carried out at the laboratories of the Seed Registration and Certification Center Directorate in Ankara.

The plant material consisted of six forage pea (*Pisum sativum* L. var. arvense) cultivars: Uysal, Özkaynak, Kurtbey, Ürünlü, Taşkent, and Nany.

For the laboratory experiments, germination boxes, 0.9 mm-diameter washed sand, a calibrated germination chamber, sterilization equipment, an electronic moisture meter, EC meter (electrical conductivity), precision balance, potassium chloride (KCl), and standard laboratory consumables were used. Prior to experimentation, seed lots were evaluated for their physical properties. Thousand seed weights were determined, and seed moisture contents were adjusted to fall within the range of 10–14% before storage and testing.

The adjusted moisture levels ensured comparability across the cultivars in subsequent viability and vigor tests. These values were used as baseline data for evaluating the effects of storage on seed performance (Table 1).

### 2.1. Climatic Characteristics of the Research Area

Climatic data for the experimental site (Manisa/Beydere) were obtained from the Turkish State Meteorological Service. The data, including monthly average temperatures, total precipitation, and relative humidity values, are presented in Table 2.

As shown in the table, the long-term average temperature(1991–2022) in the region is **12.3 °C**, while the average total precipitation is approximately 86.1 mm. The data indicate that the 2021–2022 growing season closely aligned with the long-term climatic trends, suggesting normal environmental conditions during the study period.

This climate is typical of a Mediterranean-type transitional zone, characterized by mild, rainy winters and warm, relatively dry springs—suitable for cool-season forage legumes like forage pea.

These climatic data are crucial for interpreting germination, vigor, and field emergence results, as temperature and moisture are key determinants in seed metabolic activity and seedling establishment.

### 2.2. Plant Material and Seed Preparation

The forage pea (*Pisum sativum* L. var. arvense) seeds used in the study were harvested in 2021. Six cultivars—Uysal, Özkaynak, Kurtbey, Ürünlü, Taşkent, and Nany—were obtained after harvest. The seeds were not subjected to any pre-treatment. Moisture content was adjusted to between 10% and 14%, and the seeds were transferred to moisture-stable paper envelopes and stored to prevent moisture fluctuations.

### 2.3. Experimental Design

Cold storage conditions were maintained at 5 °C and approximately 60% relative humidity (RH). The study was conducted in two stages:

- **Stage I:** Initial laboratory analyses conducted without storage treatment.
- **Stage II:** Post-storage assessments after seeds were kept for three months under room temperature and controlled storage (5 °C, 60% RH).

### 2.4. Laboratory Tests

#### 2.4.1. Standard Germination Test

**Figure 1:**
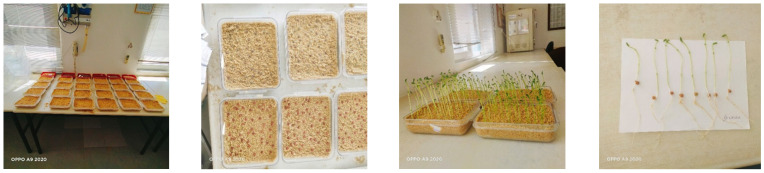
Germination tests and seedling characteristics.

Conducted in accordance with the rules of the International Seed Testing Association (ISTA, 2022) [17]. For each cultivar, 100 seeds per replicate were tested with four replications. Seeds were sown in 20 × 16 × 5 cm covered germination boxes containing sterilized (150 °C, 2 h) 0.9 mm sand at 60% moisture. The seeds were covered with 1–2 cm of the same sand, and boxes were incubated in a germination chamber at 20 ± 1 °C. Germination speed was calculated on the fifth day, and germination percentage on the eighth day (Figure 1).

#### 2.4.2. Electrical Conductivity Test

Following ISTA protocols, 50 seeds per four replications were weighed (precision 0.01 g), placed in 250 mL of deionized water in 500 mL Erlenmeyer flasks, and incubated at 20 °C for 24 h. An EC meter (HI 9033), calibrated with 0.01 M KCl (1273 µS/cm at 20 °C), was used to measure electrolyte leakage. Results were expressed in µS/cm/g.

### 2.5. Field Emergence Test

The field trial was established on 17 November 2021 and completed on 12 May 2022, using a randomized complete block design with three replications. Each plot measured 5 m × 1.5 m (7.5 m^2^) with six rows and 25 cm row spacing. A total of 750 seeds were sown per plot using a precision seed drill. Sprinkler irrigation was applied immediately after sowing to ensure uniform emergence. Climatic data were recorded throughout the trial.

### 2.6. Measured Traits

- **Total germination after 5 days (%):** Early germination percentage (%) was recorded on day 5.
- **Total germination after 8 days (%):** Final germination percentage (%) on day 8. **Electrical Conductivity (µS/cm/g):** Index of seed membrane integrity.
- **Field Emergence (%):** Ratio of emerged seedlings in field plots.
- **Plant Height (cm):** Measured from soil surface to plant apex in 10 randomly selected plants per plot.
- **Days to Flowering (days):** Days from sowing until flowering reached a specific threshold.
- **Green Forage Yield (kg/ha):** Based on 4 m^2^ harvested area per plot, extrapolated to per hectare (ha).
- **Dry Matter Yield (kg/da):** 500 g subsample dried at 70 °C for 48 h until constant weight, extrapolated.
- **Seed Moisture Content (%):** Measured with an electronic seed moisture tester.
- **Thousand Seed Weight (g):** Calculated from the mean weight of four samples of 100 seeds × 10.

### 2.7. Statistical Analysis

All data were subjected to analysis of variance (ANOVA) using the JUMP statistical software (JMP Pro 16). A factorial arrangement in a randomized complete block design was applied. Significant differences between means were determined using the LSD multiple comparison test at *p* < 0.05.

## 3. Results and Discussion

### 3.1. Electrical Conductivity Test Results (µs cm^−1^g^−1^)

**Figure 2:**
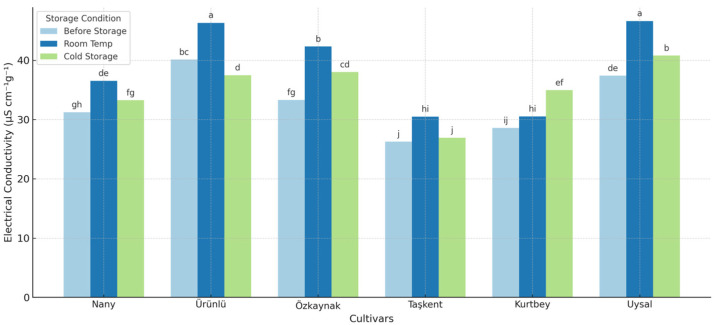
Electrical conductivity values (µS cm^−1^g^−1^) of forage pea cultivars under different storage conditions. Different lowercase letters above the bars indicate significant differences between cultivar means according to LSD test (*p* < 0.05).

The electrical conductivity (EC) test results indicated significant differences (*p* < 0.05) among forage pea cultivars and storage conditions in terms of seed vigor (Figure 2). The mean EC values across cultivars were highest under room temperature storage conditions (38.81 µS cm^−1^g^−1^), followed by cold storage (35.24 µS cm^−1^g^−1^), and the lowest values were observed in seeds prior to storage (32.82 µS cm^−1^g^−1^). This trend suggests that storage conditions, particularly room temperature, can negatively affect seed membrane integrity and vigor, leading to greater electrolyte leakage.

Among the cultivars, Ürünlü and Uysal exhibited significantly higher EC values across storage conditions, with average values of 41.31 and 41.61 µS cm^−1^g^−1^, respectively, indicating reduced seed vigor. In contrast, Taşkent showed the lowest EC values in all storage conditions, with a mean of 27.90 µS cm^−1^g^−1^, reflecting superior membrane stability and seed quality. The interaction between cultivar and storage period was also statistically significant, emphasizing that seed response to storage is cultivar-dependent.

These findings are consistent with the principle that higher EC values are associated with greater membrane damage and lower seed vigor [17]. Similar results have been reported by [18], who observed elevated EC values in legume seeds stored under suboptimal conditions, leading to decreased emergence and seedling performance. In particular, cold storage at 5 °C and 60% relative humidity appeared to mitigate the increase in EC values compared to ambient room storage, highlighting its effectiveness in preserving seed quality.

### 3.2. Total Germination After 5 Daysof Forage Pea Cultivars Results

**Figure 3:**
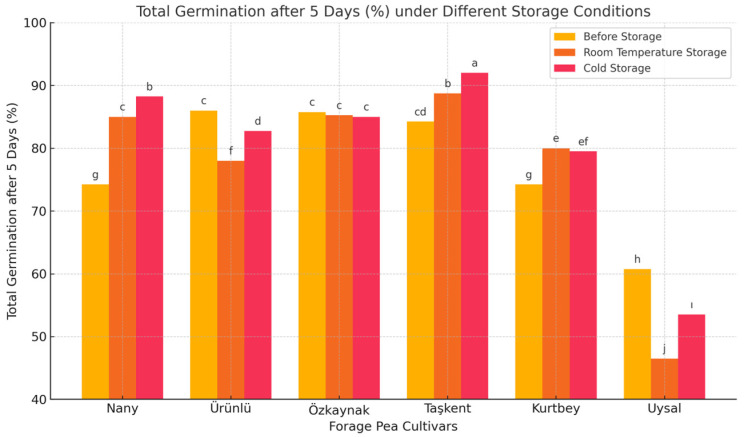
Total germination after 5 days (%) of forage pea cultivars in different storage periods. Different lowercase letters above the bars indicate significant differences between cultivar means according to LSD test (*p* < 0.05).

Figure 3 compares the total germination after 5 days (%)of six different forage pea cultivars under three storage conditions: Before Storage, Room Temperature Storage, and Cold Storage.

Cold storage generally maintained the highest total germination percentage, indicating that low-temperature conditions are more effective in preserving seed viability.

The Taşkent cultivar showed the highest total germination performance across all periods. Notably, it achieved a 92% total germination percentage under cold storage and was statistically grouped as “a,” indicating superiority.

The Uysal cultivar exhibited the lowest total germination percentages, with a significant decrease after room temperature storage. This suggests that Uysal is more sensitive to storage-related stress.

Nany and Kurtbey cultivars showed similar moderate-to-low total germination percentage. Özkaynak and Ürünlü cultivars performed well with relatively high and stable total germination percentage across different storage periods. The LSD (Least Significant Difference) values indicate statistically significant differences among cultivars and storage periods (LSD for Period: 5.59; Cultivar: 3.95; Interaction: 2.28). The letter groupings (e.g., a, b, c…) reflect statistical significance: values sharing the same letter are not significantly different, while different letters denote significant differences. Seed total germination capacity varies by cultivar, but cold storage generally yields the best results. Therefore, cold storage is recommended for long-term seed preservation. The Taşkent and Özkaynak cultivars stand out for their high and consistent total germination performance.

Seed germination is a critical indicator of seed viability and vigor, and it is directly influenced by genetic factors and storage conditions. The findings of this study clearly indicate that cold storage is more effective in preserving the total germination potential of forage pea (*Pisum sativum* L. var. arvense) seeds compared to room temperature storage. This result aligns with previous studies suggesting that low temperatures slow down metabolic activities and reduce seed deterioration over time [19,20].

Among the cultivars tested, Taşkent exhibited the highest total germination percentage across all storage periods, particularly under cold storage conditions. This suggests a strong genetic tolerance to storage stress, making it a promising candidate for long-term seed conservation and organic agriculture systems. In contrast, the Uysalcultivar showed significantly lower total germination percentage, especially after room temperature storage, indicating that it is more vulnerable to unfavorable storage conditions. This cultivar may require improved seed handling practices or shorter storage durations to maintain acceptable total germination levels.

Özkaynak and Ürünlü cultivars showed stable and relatively high total germination across all storage conditions, demonstrating resilience that could be beneficial in various ecological settings. The statistical analysis confirmed significant differences among cultivars, storage methods, and their interactions, supporting the necessity to tailor storage protocols based on cultivar-specific responses.

Overall, this study emphasizes the importance of selecting genetically resilient cultivars and employing proper storage strategies, particularly cold storage, to maintain seed viability and ensure successful crop establishment.

### 3.3. Total Germination After 8 Days (%) Results

**Figure 4:**
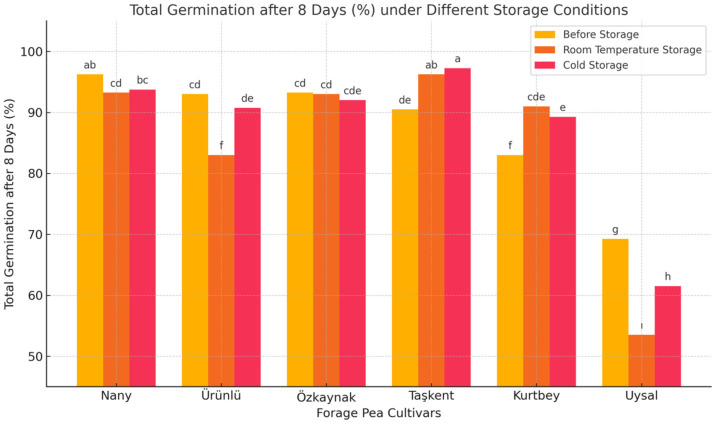
Total germination after 8 (%) of forage pea varieties at different storage periods (Before storage, Room temperature, Cold storage). Different letters indicate statistically significant differences (*p* < 0.05) according to LSD test.

Figure 4 illustrates the total germination after 8 daysof six forage pea varieties under three storage conditions. Notable differences were observed between storage methods and among varieties. The cold storage condition generally maintained higher total germination after 8 days compared to room temperature.

Storage conditions had a significant effect on the total germination after 8 days of forage pea seeds. In general, seeds stored in cold conditions preserved their viability better than those stored at room temperature. This result aligns with previous studies that emphasized the importance of low temperatures in reducing seed deterioration during storage.

Among the varieties, ‘Taşkent’ and ‘Nany’ showed the highest total germination after 8 daysacross all storage conditions, indicating superior seed longevity and potential adaptability to different storage environments. Conversely, ‘Uysal’ exhibited the lowest total germination values, especially after room temperature storage, suggesting that it may be more sensitive to unfavorable storage conditions.

The observed variety × storage interaction was statistically significant, implying that each variety responded differently to the storage treatments. These findings suggest that both genotype and storage environment should be considered in seed conservation and storage planning.

In practical terms, cold storage is recommended to maintain seed quality in forage peas, especially for varieties with lower innate storage resilience. Selecting varieties like ‘Taşkent’ or ‘Nany’ may further enhance seed viability over time.

Storage temperature significantly affected the total germination after 8 days of forage pea (*Pisum sativum* L. var. arvense) seeds. In this study, cold storage preserved seed viability more effectively than room temperature conditions. These results are consistent with findings from [18,19], who reported that low-temperature environments help reduce the metabolic activity and oxidative stress that cause seed deterioration during storage.

Varietal differences were also prominent. ‘Taşkent’ and ‘Nany’ maintained high total germination after 8 days across all storage conditions, indicating superior seed quality and storage stability. On the other hand, ‘Uysal’ was more sensitive to storage conditions, especially under room temperature, exhibiting a significant decline in viability. Similar genotype-dependent responses to storage have been reported by [21,22], who emphasized the influence of genetic makeup on seed longevity.

The significant variety × storage interaction observed in this study suggests that not all varieties respond similarly to storage environments. This underlines the importance of selecting suitable genotypes for long-term seed storage programs, especially under suboptimal conditions. As proposed by [12], seed storage potential is largely influenced by both inherent physiological properties and external storage conditions.

Comparable results have also been reported in similar studies. For example, ref. [23] observed germination spread ranging between 89.6% and 98.6% in forage pea seeds of different colors. Ref. [24] found that zinc applications increased total germination after 8 to between 87% and 97.3%, while boron applications resulted in germination values ranging from 80% to 95.3%. Likewise, ref. [25] reported that under salt stress conditions, total germination after 8 in forage pea seeds varied between 34.5% and 98.5%. In another study, ref. [26] reported germination values between 89% and 95% in soybean seeds treated with different boron concentrations. These findings suggest that the total germination after 8 values observed in our study islargely consistent with those reported in the literature, and no significant deviations were found.

Overall, cold storage is recommended to maintain seed viability in forage pea, particularly for varieties like ‘Uysal’ with lower innate storage resilience. For seed producers and breeders, selecting storage-tolerant varieties such as ‘Taşkent’ may provide better outcomes in terms of total germination and vigor preservation.

### 3.4. Field Emergence Rates (%) Results

**Figure 5:**
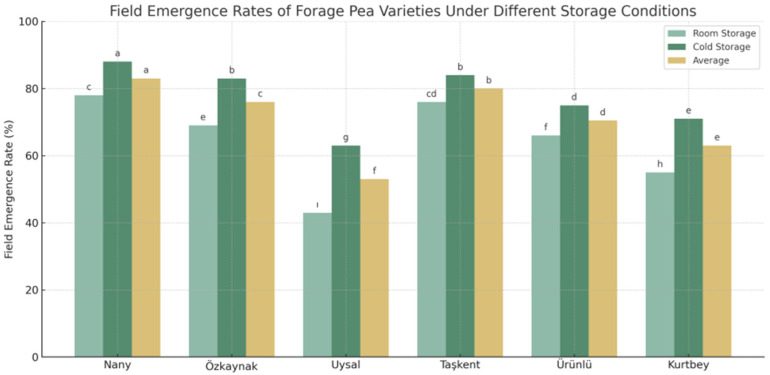
Field emergence rates (%) of forage pea varieties under different storage conditions. Different lowercase letters above the bars indicate significant differences between cultivar means according to LSD test (*p* < 0.05).

Figure 5 illustrates the field emergence percentages of six forage pea (*Pisum sativum* L. var. arvense) varieties under two different storage conditions: room temperature and cold storage. Additionally, the average emergence values across both storage types are presented. The statistical groupings indicated by letters above the bars reveal significant differences among the varieties and treatments (LSD, *p* < 0.05).

The highest field emergence rate under cold storage conditions was observed in the ‘Nany’ variety (88%), which was significantly superior and labeled as group “a.” Under room temperature storage, ‘Nany’ again performed well (78%), while ‘Uysal’ had the lowest emergence rate (43%) and was statistically grouped as “ı” Cold storage consistently improved emergence across all varieties, with average values increasing from 64.5% (room storage) to 77.3% (cold storage).

The results clearly indicate that cold storage significantly enhances seed field emergence compared to room temperature storage. This aligns with previous findings that suggest cool, controlled storage environments help maintain seed viability and reduce metabolic deterioration [27,28].

Among the evaluated varieties, ‘Nany’ and ‘Taşkent’ showed the most consistent and high emergence rates, suggesting their greater tolerance to post-harvest storage conditions. Conversely, ‘Uysal’ and ‘Kurtbey’ displayed lower emergence performance, particularly under room conditions, indicating their sensitivity to storage-induced viability loss.

The interaction effect (Variety × Storage Condition) was also significant (LSD = 2.02), showing that some varieties respond more strongly to storage treatments than others. This finding underscores the importance of selecting both storage strategies and variety types carefully in seed production systems.

These results are particularly relevant for forage pea production in regions with limited cold storage infrastructure, as they highlight the need to prioritize varieties like ‘Nany’ that retain higher viability even when stored under suboptimal conditions.

## 4. Agronomic Evaluation of Forage Pea Varieties Under Different Storage Conditions

The following Table 3 presents a comparative analysis of forage pea varieties grown under two storage conditions (room temperature and cold storage). Key agronomic traits including plant height, flowering time, green forage yield, and dry forage yield are presented along with averages and statistical information. The present study demonstrated that seed storage conditions significantly influenced plant growth and forage yield traits in Forage pea (*Pisum sativum* L. var. arvense). Notably, cold storage conditions resulted in increased plant height, earlier flowering, and improved forage yields compared to room temperature storage, aligning with previous findings on legume crops.

### 4.1. Plant Height

The plant height of the Forage pea varieties significantly varied under both room and cold storage conditions. Among all varieties, ‘Özkaynak’ recorded the greatest average plant height (113.8 cm), followed by ‘Taşkent’ (107.2 cm), whereas ‘Kurtbey’ exhibited the shortest plants (90 cm). Overall, cold storage slightly improved plant height compared to room conditions. According to LSD values, differences among varieties were statistically significant (LSD (Variety) = 2.53 cm) and also influenced by storage conditions (LSD (VxS) = 1.84 cm). The coefficient of variation (CV) was relatively low (1.39%), indicating high data reliability.

### 4.2. Days to Flowering

The flowering time varied considerably among varieties. ‘Nany’ took the longest to flower (average 145.66 days), while ‘Kurtbey’ was the earliest maturing variety (121.33 days). Interestingly, cold storage slightly reduced the days to flowering in most varieties, indicating that it might enhance earliness. However, the LSD values showed that the variety and storage interaction (VxS) was not significant, indicating a uniform response across environments.

### 4.3. Green Forage Yield

Green forage yield ranged from 11,058 kg/ha (‘Taşkent’ under room conditions) to 15,911 kg/ha (also ‘Taşkent’ under cold conditions), suggesting a strong varietal response to cold storage. ‘Ürünlü’ showed the highest average green forage yield (14,003 kg/ha), whereas ‘Kurtbey’ had the lowest (13,033 kg/ha). The average yield increased notably under cold storage (14,933 kg/ha) compared to room conditions (11,896 kg/ha), highlighting the positive effect of cold storage on fresh biomass accumulation. These differences among storage conditions were statistically significant, while varietal differences were not significant for green forage yield.

### 4.4. Dry Forage Yield

Dry forage yield followed a similar trend to green forage. The highest dry yield was recorded in ‘Ürünlü’ (3124–4110 kg/ha), followed by ‘Nany’ and ‘Uysal’. As with green yield, cold storage enhanced dry matter accumulation in all varieties. The average dry yield under cold conditions (4154 kg/ha) was significantly higher than under room storage (2979 kg/ha). Again, the differences among varieties were not statistically significant, as indicated by the LSD values.

The results indicate that cold storage has a generally positive effect on plant height, green and dry forage yield, and slightly accelerates flowering in Forage pea varieties. Although varietal differences in yield were not statistically significant, consistent trends suggest that ‘Ürünlü’, ‘Taşkent’, and ‘Nany’ may be more suitable for high biomass production under improved seed storage conditions.

The shorter flowering duration observed under cold storage implies potential benefits in escaping terminal drought stress, especially in regions with shorter growing seasons. The varieties ‘Kurtbey’ and ‘Ürünlü’, being early maturing, could be useful in such environments. On the other hand, the tall and high-yielding varieties like ‘Özkaynak’ and ‘Taşkent’ may be better suited for areas with longer vegetative periods.

Overall, cold storage appears to improve early vigor and biomass productivity, possibly by preserving seed viability and physiological quality. This study highlights the importance of seed storage conditions on field performance and suggests that selection of appropriate varieties combined with optimized storage methods can enhance forage production efficiency.

### 4.5. Effect of Storage Conditions on Plant Height and Flowering

The significantly taller plants observed under cold storage (average 103.53 cm) compared to room conditions (101.36 cm) suggest that seed vigor was better maintained, resulting in improved early growth and biomass accumulation. These findings are consistent with the work of [29], who reported that optimal seed storage conditions preserve enzymatic activity and hormonal balance, supporting better seedling establishment and vegetative development in legumes.

Additionally, cold storage led to slightly earlier flowering across most varieties, with the average days to flowering reduced from 134.00 days (room) to 131.33 days (cold). Ref. [30] found that lower storage temperatures contributed to improved germination and uniform seedling emergence, which often correlates with earlier phenological development. Early flowering is agronomically advantageous, especially in Mediterranean climates, as it allows crops to escape terminal drought stress [31].

### 4.6. Forage Yield Performance

Both green and dry forage yields were higher under cold storage, with an average green yield of 14,933 kg/ha and dry yield of 4154 kg/ha, compared to 11,896 kg/ha and 2979 kg/ha, respectively, in room-stored seeds. The variety ‘Ürünlü’ consistently performed well in both green and dry forage production, suggesting genetic potential combined with enhanced seed physiological quality.

These results are supported by [32], who highlighted the critical role of seed quality in determining field emergence and biomass yield in forage legumes. Similarly, ref. [33] reported that higher seedling vigor, achieved through cold storage, leads to better canopy development and forage accumulation.

While the differences among varieties for forage yield were not statistically significant, the overall trend showed that some varieties (‘Ürünlü’, ‘Taşkent’, ‘Nany’) responded more positively to cold storage, indicating a variety-dependent response to storage conditions. Ref. [34] emphasized the importance of considering genotype × environment interactions, particularly in studies involving seed handling and crop performance.

### 4.7. Implications for Agronomic Practice

These findings suggest that cold storage of Forage pea seeds can significantly enhance forage productivity and phenological development, especially under organic or low-input systems where seedling vigor is crucial. The results underline the importance of maintaining high seed quality through appropriate post-harvest handling and storage protocols.

Furthermore, the selection of early-flowering and high-biomass varieties like ‘Ürünlü’ and ‘Taşkent’ could provide adaptive advantages in semi-arid and Mediterranean environments, aligning with the conclusions of [35] who emphasized the value of early maturity and forage potential in vetch breeding programs.

## 5. Conclusions

This study demonstrated that storage conditions have a significant impact on the seed quality and agronomic performance of forage pea (*Pisum sativum* L. var. arvense) cultivars. Cold storage at 5 °C and 60% relative humidity effectively preserved seed vigor, as evidenced by lower electrical conductivity values, higher germination rates, and improved field emergence compared to room temperature storage. Among the cultivars tested, Taşkent, Nany, and Özkaynak showed superior seed viability and vigor across all storage periods, while Uysaland Kurtbey were more vulnerable to deterioration, particularly under ambient conditions.

The positive influence of cold storage extended to field performance, resulting in taller plants, earlier flowering, and higher green and dry forage yields. Notably, Ürünlü and Taşkent cultivars produced the highest biomass yields under cold storage, highlighting their potential for high productivity when optimal seed handling practices are employed.

The significant interactions between cultivar and storage conditions underscore the importance of considering genotype-specific responses when developing seed storage strategies. These findings emphasize that maintaining seed quality through appropriate post-harvest storage practices, particularly cold storage, is essential for ensuring successful crop establishment, maximizing forage yield, and supporting sustainable agricultural production in Mediterranean and similar agro-ecological zones.

Future studies may focus on the long-term effects of different storage durations and the integration of biochemical and molecular indicators to further elucidate the mechanisms behind varietal differences in seed longevity and field performance.

## Figures and Tables

**Table 1:** Characteristics of forage pea cultivars used in the experiment.

Forage Pea Cultivar	Thousand Seed Weight (g)	Pre-Storage Seed Moisture (%)	Post-Storage Seed Moisture (%)
**Uysal**	160.80	13.4	12.6
**Özkaynak**	141.60	11.4	13.2
**Taşkent**	146.40	11.0	10.6
**Kurtbey**	194.60	10.6	10.4
**Ürünlü**	123.38	11.6	13.8
**Nany**	118.00	11.7	10.8

**Table 2:** Monthly climatic data for the experimental area (Manisa/Beydere).

Month	Avg. Temp (°C) 2021–2022	Avg. Temp (°C) 1991–2022	Total Precip. (mm) 2021–2022	Total Precip. (mm) 1991–2022	Relative Humidity (%) 2021–2022	Relative Humidity (%) 1991–2022
October	18.0	17.8	50.4	51.8	67	64
November	12.0	12.1	86.8	88.9	76	73
December	8.0	8.1	135.4	138.2	81	80
January	7.0	6.6	127.2	129.0	82	79
February	7.0	7.9	105.4	107.7	77	75
March	10.0	10.5	77.1	78.4	73	70
April	15.0	15.1	53.6	55.5	68	65
May	20.0	20.3	38.5	39.3	59	57
Mean	12.0	12.3	84.3	86.1	73	70

*Source: [16].*

**Table 3:** Agronomic traits of forage pea varieties under different storage conditions.

Varieties	Plant Height			Flowering Time			Green Forage			Dry Forage		
	Room (cm)	Cold (cm)	Avg.	Room (Days)	Cold (Days)	Avg.	Room (kg/ha)	Cold (kg/ha)	Avg.	Room (kg/ha)	Cold (kg/ha)	Avg.
Nany	100 f	104 cd	102 c	146.33 a	145	145.66 a	11,862	15,244	13,553	3065	4295	3680
Özkaynak	113 a	114.6 a	113.8 a	138.33 b	136.33	137.33 b	11,591	14,889	13,239	2964	4057	3510
Uysal	101.2 ef	103 de	102.2 c	136 c	132	134.00 c	12,529	13,822	13,175	3065	4011	3538
Taşkent	106.3 bc	108 b	107.2 b	134 c	131	132.50 d	11,058	15,911	13,484	2798	4415	3606
Ürünlü	100 f	101.6 def	100.8 c	127 d	123.33	125.16 e	12,762	15,244	14,003	3124	4110	3617
Kurtbey	88 h	92 g	90 d	122.0 e	120.33	121.33 f	11,578	14,489	13,033	2861	4036	3448
**Average**	101.36 b	103.53 a		134.00 a	131.33 b		11,896 b	14,933 a		2979 b	4154 a	
**LSD** **(Variety)**	2.53			3.35			Not Sig.			Not Sig.		
**LSD (VxS)**	1.84			Not Sig.			Not Sig.			Not Sig.		
**CV (%)**	1.39			0.81			126.1			106.0		

Different lowercase letters in the table indicate significant differences between cultivar means according to LSD test (*p* < 0.05).

## Data Availability

The data presented in this study are available within the article.
